# Prebiliary Right Hepatic Artery Resulting in Common Hepatic Duct Compression and Subsequent Intrahepatic Stone Formation: Myth or Reality?

**DOI:** 10.1155/2014/403104

**Published:** 2014-01-09

**Authors:** Vanessa Marron Mendes, Haydar A. Nasser, Georges Bou Nassif, Ali Choukr

**Affiliations:** ^1^Université Libre de Bruxelles (ULB), 1000 Brussels, Belgium; ^2^Lebanese University, Beirut 961, lebanon

## Abstract

The vascular anatomy of the liver is subjected to many variations. Aberrant hepatic artery is not an uncommon finding during visceral surgery; however, topographic variations are less reported in the literature. Prebiliary artery crossing anteriorly to the common hepatic duct was firstly reported in 1984. We present here a case of a 52-year-old lady who presented with obstructive jaundice and right upper quadrant pain. Paraclinical investigations were consistent with intrahepatic stones and a benign stricture on the CBD. During surgery, a prebiliary right hepatic artery compressing the CHD was noted. The liver pedicle was dissected and a hepaticojejunostomy was performed that resulted in a good outcome after 24 months of followup.

## 1. Introduction 

In the 20th century, Michels [1] and Hiatt et al. [2] proposed internationally recognized schemes for the classification of the hepatic arterial anatomy with its variations. In a retrospective study, 604 selective angiographies showed that the conventional vascular anatomy of the liver was found in 79.1% of individuals [3]. Normally, the hepatic artery courses between the extrahepatic bile duct and the portal vein. Aberrant and replaced right hepatic arteries (RHA) are well-known variants. Topographic variations, such as anterior or retroportal RHA, have been rarely described before [4–7]. The first case of Jaundice, and hepatolithiasis due to an anterior RHA pressing on the CBD was reported in 1984. Since then, one similar case was reported [8] and the current one is to be added.

## 2. Case Report

A 52-year-old lady was referred to general surgery clinics because of 2 months history of jaundice and right upper quadrant pain. She has no previous medical history. Clinically the patient was stable hemodynamically, without any signs of sepsis. On CT-scan, the bile ducts were dilated and filled with stones. The MRCP was not of diagnostic value due to motion artifacts. The total serum bilirubin increased to 5 mg/dL from a baseline of 2.9 mg/dL. She was offered ERCP twice without diagnostic yield, and her symptoms persisted despite sphincterotomy. Alkaline phosphatase serum level was also elevated (3 times of normal). The decision was to proceed with an exploratory laparotomy in order to relieve the symptoms and establish a diagnosis. This treatment option was discussed with the patient, and she had signed the informed consent the night before the procedure.

**Figure 1:**
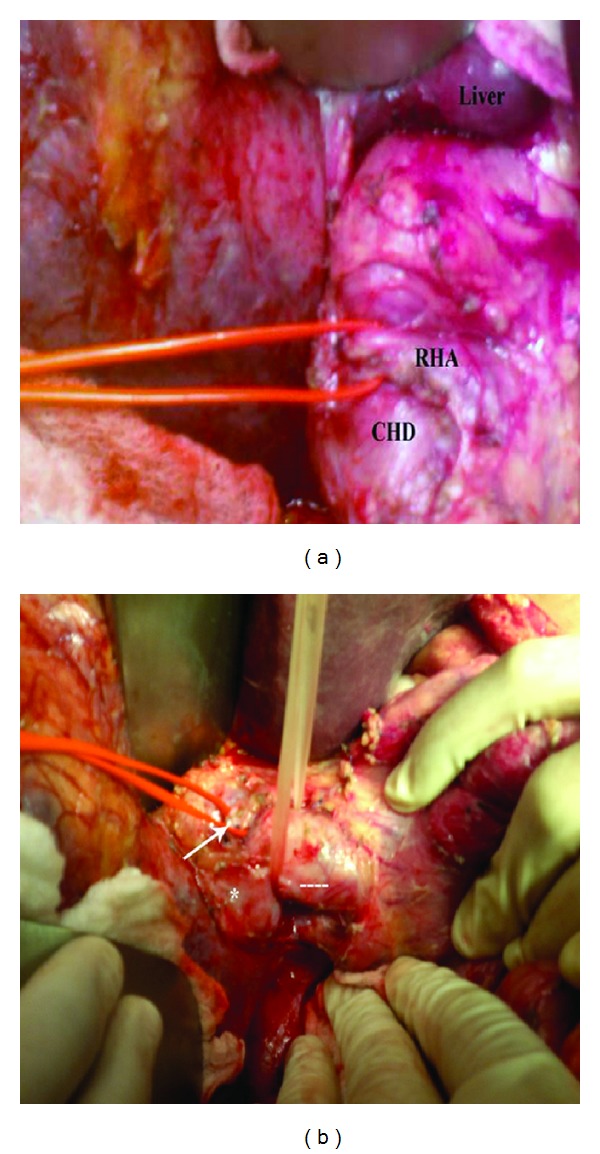
(a) RHA (red lacks) crossing anteriorly to the CHD and compressing it. RHA: right hepatic artery; CHD: hepatic duct. (b) Lateral view of the hepaticoduodenal ligament. The RHA (white arrow) crossing anteriorly to the dilated CHD (dashed line). Asterisk corresponds to the dilated right hepatic duct.

**Figure 2:**
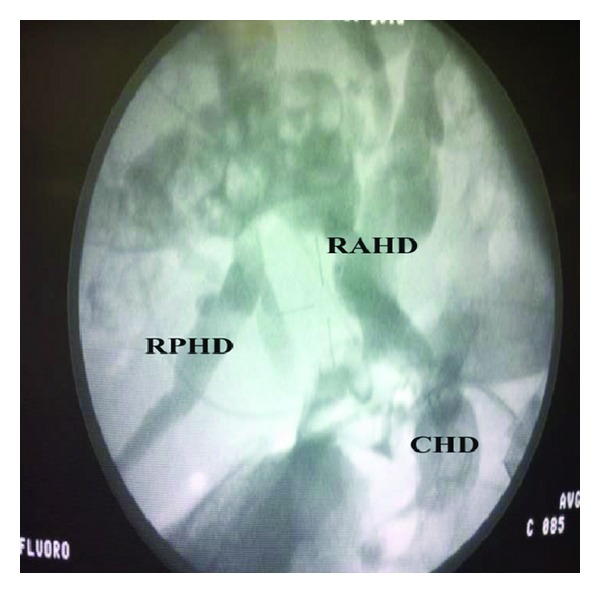
Intraoperative cholangiogram showing the dilated common hepatic duct (CHD), right posterior hepatic duct (RPHD), and the right anterior hepatic duct (RAHD).

**Figure 3:**
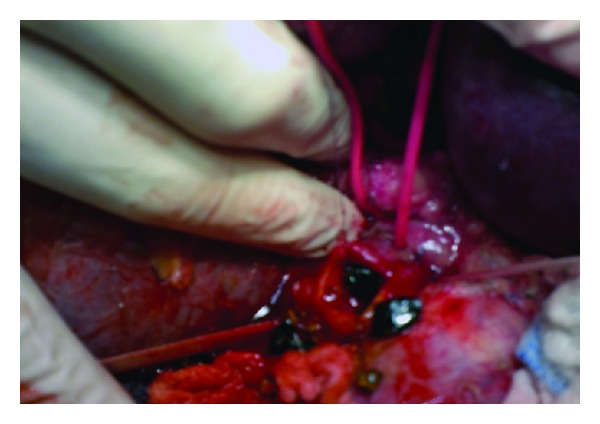
Choledochotomy and evacuation of the pigmented stones.

At laparotomy, an aberrant anterior right hepatic artery pressing on the CHD was noted. The CHD was dilated just proximal to the crossing point (Figures 1(a) and 1(b)). The artery was dissected off the CHD. An intraoperative cholangiography showed intrahepatic ducts filled with stones (Figure 2). We performed a choledochotomy to facilitate the complete evacuation of these dark-brown stones (Figure 3). A hepaticojejunostomy was then fashioned. The post-operative course was uneventful and the patient has had her symptoms completely resolved within 2 weeks. We are now at 24 months after surgery with no signs of recurrence.

## 3. Discussion

**Figure 4:**
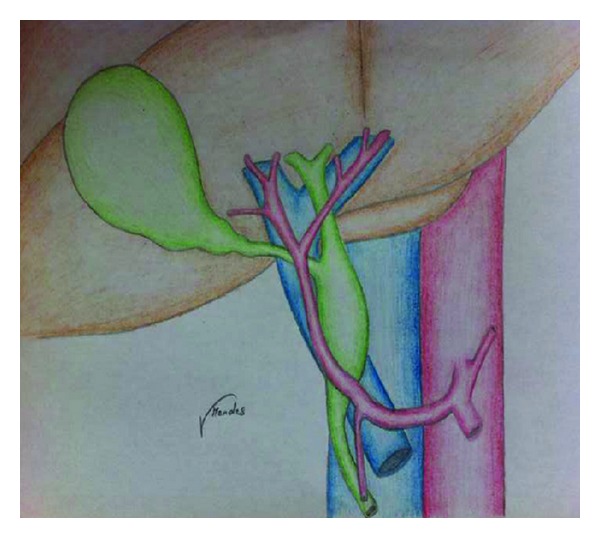
Schematic diagram illustrating the compression on the CBD exerted by the anterior aberrant RHA.

Pseudoobstruction of the CHD by a pulsatile RHA has been previously documented on MRCP and should not be misdiagnosed as obstructing periampullary tumors [10]. Usually, the absence of obstructive symptoms confirms this diagnosis. However, jaundice due to pulsatile RHA has been reported previously [5, 6, 8, 9, 11], and this is known as the right hepatic artery syndrome [5]. The RHA usually runs posteriorly to the bile duct and can result in an inflammatory impingement of the CHD [5, 6, 10]. Two authors [8, 9] have described 3 cases with this syndrome but with distinguishing features: anterior RHA and hepatolithiasis. Tsuchiya et al. [9] and Baek et al. [8] attributed jaundice and gallstone formation to an aberrant RHA running anteriorly to the CBD. Whether or not compression of the CHD has led to stones formation is questionable. The first case report was criticized by Professor G.B. Ong [9]; he stated that intrahepatic stones formation preceded the obstructive symptoms and that the stones got impacted within the CHD, at the level of the arterial crossing. However, if pulsatile compression is an acceptable theory (the right hepatic artery syndrome), then subsequent intrahepatic stone formation should also be regarded as an acceptable one; partial or complete obstruction of the CBD/CHD leads to bile stasis and later on to bacterial proliferation [12]. One experimental study showed that liver bacterial concentration was significantly elevated after bile duct ligation [13]. Bacteria can reach the liver either by translocation from the gut or through the blood [12] and result in the so called pigmented or dark-brown gallstones formation [14]; as a result of this sequence analysis, compression of the CHD by an aberrant RHA could lead to bile stasis ande then bacterial proliferation and ends with gallstone formation. In 3 reported cases of right hepatic artery syndrome [5, 6, 11], the RHA was running posteriorly to the CBD and none of the authors described intrahepatic gallstones. Another important feature in Tsuchiya et al., report [9] that is worth mention was the absence of gallstones distal to the compression site. This was also an intraoperative finding cited by Baek and colleagues [8].  Table 1 summarizes the treatment modalities adopted in each case. In our case, choledochotomy followed with Roux-en-Y hepaticojejunostomy resulted in a good outcome. To the best of our knowledge, this is the 4th case in the literature where an anterior crossing RHA results in biliary stasis and hepatolithiasis. The dark-brown aspect of the stones observed in our case and elsewhere [9] is highly suggestive of their infectious nature. This was probably due to the cord-like effect that was exerted by the aberrant RHA on the CBD (Figure 4).

## Figures and Tables

**Table 1:** Different treatment modalities adopted in patients with hepatolithiasis due to right hepatic artery syndrome.

Author [reference]	*n*	Operations	Stones aspect	Postoperative course
Baek et al. [8]	1	Hepaticojejunostomy	N/A	Uneventful

Tsuchiya et al. [9]	2	Hepaticojejunostomy in case 1	Pigmented	Case 1: uneventful
		Left partial hepatectomy + hepatico-jejunostomy in case 2	Pigmented	Case 2: patient died on day 53 after operation due to bleeding jejunal ulcer at the site of hepatico-jejunostomy

(*n*): number of patients; N/A: not available.
